# Analysis of renewable energy consumption and economy considering the joint optimal allocation of “renewable energy + energy storage + synchronous condenser”

**DOI:** 10.1038/s41598-023-47401-4

**Published:** 2023-11-21

**Authors:** Zesen Wang, Qi Li, Shuaihao Kong, Weiyu Li, Jing Luo, Tianxiao Huang

**Affiliations:** Electric Power Research Institute State Grid Jibei Electric Power Company Limited, Beijing, 100045 China

**Keywords:** Engineering, Energy science and technology

## Abstract

As renewable energy becomes increasingly dominant in the energy mix, the power system is evolving towards high proportions of renewable energy installations and power electronics-based equipment. This transition introduces significant challenges to the grid’s safe and stable operation. On the one hand, renewable energy generation equipment inherently provides weak voltage support, necessitating improvements in the voltage support capacity at renewable energy grid points. This situation leads to frequent curtailments and power limitations. On the other hand, the output of renewable energy is characterized by its volatility and randomness, resulting in substantial power curtailment. The joint intelligent control and optimization technology of “renewable energy + energy storage + synchronous condenser” can effectively enhance the deliverable capacity limits of renewable energy, boost its utilization rates, and meet the demands for renewable energy transmission and consumption. Initially, the paper discusses the mechanism by which distributed synchronous condensers improve the short-circuit ratio based on the MRSCR (Multiple Renewable Energy Station Short-Circuits Ratio) index. Subsequently, with the minimum total cost of system operation as the optimization objective, a time-series production simulation optimization model is established. A corresponding optimization method, considering the joint configuration of “renewable energy + energy storage + synchronous condenser,” is proposed. Finally, the effectiveness of the proposed method is verified through common calculations using BPA, SCCP, and the production simulation model, considering a real-world example involving large-scale renewable and thermal energy transmission through an AC/DC system. The study reveals that the joint intelligent control and optimization technology can enhance both the sending and absorbing capacities of renewable energy while yielding favorable economic benefits.

## Introduction

Currently, the large-scale proliferation of renewable energy in China is predominantly located in the northwest, north, and northeast regions. These areas are geographically opposite to the load centers, requiring the UHV AC/DC power grid for transmitting this renewable energy. As of June 2022, Qinghai Province stands out with 90% of its installed capacity coming from clean energy, and 61.8% specifically from renewable sources, making it a leader in renewable energy adoption within China. The Qinghai-Henan ± 800 kV ultra-high voltage direct current project serves as the nation’s first ultra-high voltage channel purpose-built for clean energy export, delivering around 40 billion kilowatt-hours of clean electricity from Qinghai to Henan annually. With the ongoing escalation in the scale and penetration rates of renewable energy installations, the power system is evolving towards having high proportions of renewable energy and power electronics-based equipment. This development introduces significant challenges for maintaining power balance and stability in the grid. On one hand, renewable energy generation equipment offers limited voltage support, making the grid connection points less robust. This increases the likelihood of large-scale disconnections during voltage fluctuations, compelling the grid operation departments to curtail the output from renewable energy stations. On the other hand, the inherently variable and random output of renewable energy leads to gaps in its power generation curve. For renewable energy investors, minimizing these gaps is crucial for optimizing economic returns.

The renewable energy gathering area has a large installed capacity, lacks supporting power supply, and has poor resistance to fault disturbance and impact. After a power grid fault, the transient over-voltage at the end of the renewable energy unit is too high, and in severe cases, a large-scale chain disconnection accident of the renewable energy unit may occur. Therefore, it is currently necessary to limit the output of renewable energy stations in operation to ensure the safe and stable operation of the system. This has led to long-term limitations in renewable energy generation, and the transmission capacity of ultra-high voltage projects has not been fully utilized. The synchronous condenser can not only play the role of dynamic reactive power reserve in the system, but also provide voltage support for the entire process of sub transient, transient, and steady-state for renewable energy power plants^1,2^. Configuring synchronous condensers in renewable energy stations is currently the most effective measure to improve the system’s voltage support capacity^3–6^ and suppress transient over-voltage^7,8^. However, the research focus of existing literature on the configuration of synchronous condensers is mostly on fixed capacity and site selection^9,10^, and there is little research on the effectiveness and economic benefits of synchronous condensers in promoting renewable energy consumption.

Faced with the demand for renewable energy consumption scenarios, energy storage technology has developed rapidly. As a flexible regulation resource, the spatiotemporal transfer characteristics of energy storage are of great significance for the consumption of renewable energy. According to different access locations, it can be divided into energy storage on the generation side^11,12^, grid side^13^, and user side^14^. According to different types, it can be divided into electrochemical energy storage^15^, hydrogen energy storage^16^, pumped storage^17–19^, etc. Reference^17^points out that the combination of renewable energy and pumped hydro energy storage reduces energy dependence compared with a system without storage to satisfy the required electricity demand. Reference^19^ provides the joint optimal control of pumped storage facility + wind energy + solar photovoltaic + thermal energy system under the framework of optimal power flow. The results show that pumped storage facilities can effectively promote the consumption of renewable energy. However, when the renewable energy generation is limited, the role of energy storage will be greatly weakened.

In summary, to genuinely enhance the efficiency of renewable energy utilization and promote its consumption, it is crucial to simultaneously focus on raising the output limit and suppressing output fluctuations of renewable energy. Firstly, this paper explores the mechanism by which distributed synchronous condensers improve the short-circuit ratio, based on the MRSCR (Multiple renewable energy station short-circuits ratio) index definition. Secondly, an optimization model for time-series production simulation is developed, targeting the minimization of the total system operation cost. A method for joint optimization configuration of “renewable energy + energy storage + synchronous condenser” is also proposed. Lastly, an actual case study involving large-scale renewable and thermal energy transmitted through AC/DC lines in northern regions serves as a basis for the joint calculations using BPA, SCCP, and the production simulation model. The paper quantitatively evaluates the impact of the “renewable energy + energy storage + synchronous condenser” approach on renewable energy consumption capacity and assesses the economic feasibility of this mode.

## Multiple renewable energy station short-circuit ratio lifting method

### MRSCR

Various methods exist to build short-circuit ratio (SCR) indicators^20–22^. The percentage of system short-circuit capacity to electrical equipment capacity is the short-circuit ratio. References^23^ and^24^ respectively proposed weighted short circuit ratio (WSCR) and composite short circuit ratio (CSCR) to reflect the voltage support strength of renewable energy grid-connected systems. With the development of the power system, the topology structure has shifted from single-feed to multi-feed. In 2016, The CIGRE B4.62 proposed an equivalent short circuit ratio (ESCR)^25^. On this basis, reference^26^ considered the interaction between various DC systems and proposed a multi-infeed integrated short circuit ratio (MISCR) based on the concept of equivalent coupled admittance. Reference^20^ considered the voltage interaction between different renewable energy stations and proposed a weighted equal short circuit ratio (WESCR) for renewable energy clusters. The critical short circuit ratio (CSCR) is used to reflect the necessary voltage support strength of the power system for power electronic equipment^27^, and is usually divided into 2 and 3 to determine the strength of the system^28^. Reference^22^ proposed the SCR-S calculated by the capacity was proposed according to the SCR concept, and the SCR-U calculated by the voltage was proposed by the relationship between the SCR and the node voltage. It was recommended to use two as the standard for dividing the strength of renewable energy grid-connected systems. Reference^21^ found through extensive simulation verification that the renewable energy station short circuit ratio (RSCR) values between 1.7 and 2.1 were the normal operating requirements of renewable energy power generation equipment.

The Multiple Renewable Energy Station Short-Circuits Ratio (MRSCR) is quantified as the ratio of the short-circuit capacity at the point of common coupling (PCC) of a specific renewable energy station to its overall equivalent output power, which takes into account the influence of neighboring renewable energy stations. Mathematically, MRSCR for the $i$-th renewable energy station is given by:

1 $$\begin{aligned} {\text {MRSCR}}_i = \frac{S_{di}}{P_{REi} + \sum _{j=1, j \ne i}^{n} \left| \frac{Z_{eqij}}{Z_{eqii}} \right| P_{REj}} \end{aligned}$$

where $S_{di}$ is the short-circuit capacity at the low-voltage side of the step-up transformer for the $i$-th renewable energy station, in MVA; $P_{REi}$ and $P_{REj}$ are the output power for the $i$-th and $j$-th renewable energy stations, respectively, in MW; and $\left| \frac{Z_{eqij}}{Z_{eqii}} \right|$ is the power conversion factor, accounting for the influence of the $j$-th renewable energy station on the $i$-th renewable energy station, which is related to the mutual impedance between them.

From the Eq. (1), it becomes evident that the pivotal factors influencing MRSCR include the short-circuit capacity at the point of common coupling (PCC) of each renewable energy station, the station’s renewable energy output, the renewable energy output from adjacent stations, and the mutual and self-impedances among these stations. An increase in the renewable energy output of either the focal station or its neighbors generally leads to a decrease in MRSCR.

Renewable energy sources like large-scale wind turbines and photovoltaic systems are primarily interfaced with the grid through power electronic converters. These converters exhibit poor fault current handling capabilities. Consequently, the recent fault contribution from a renewable energy station is inherently limited, leading to a reduction in the system’s overall short-circuit capacity and potentially compromising system stability. Low MRSCR values can trigger various adverse system conditions, including voltage fluctuations, transient over-voltages, and anomalous dynamic behaviors in converter stations-most notably, power oscillations following a DC commutation failure. Thus, keeping MRSCR within an acceptable range is crucial for ensuring the safe and stable operation of the power system.

In this study, a MSRCR value of 1.5 is selected as the baseline operating condition for the system, based on real-world engineering requirements and safety margins. A system is considered to be robust when its MSRCR value falls within the range of 1.5<MSRCR<2.5. When MSRCR>2.5, the power system is deemed capable of maintaining stable grid connections for renewable energy generation equipment with various performance characteristics. The relationship between MSRCR and the voltage support capability of the power system is detailed in Table 1.

**Table 1:** $MSRCR$ value table.

$MSRCR$ value range	Voltage support strength
$MSRCR$ < 1.5	Weak system
$MSRCR$ = 1.5	Critical stable
1.5<$MSRCR$ <2.5	Strong system
$MSRCR$>2.5	Super strong system

To sum up, on the premise that the renewable energy access point remains unchanged, MRSCR will limit the renewable energy output.

### Mechanism of distributed synchronous condenser to improve short circuit ratio

The synchronous condenser operates in parallel with the power grid and is situated at the AC side of both AC-to-DC and DC-to-AC converters. During system faults, the synchronous condenser provides substantial instantaneous reactive power and exhibits short-term overload capacity, making it uniquely advantageous for dynamic reactive power compensation.

**Figure 1:**
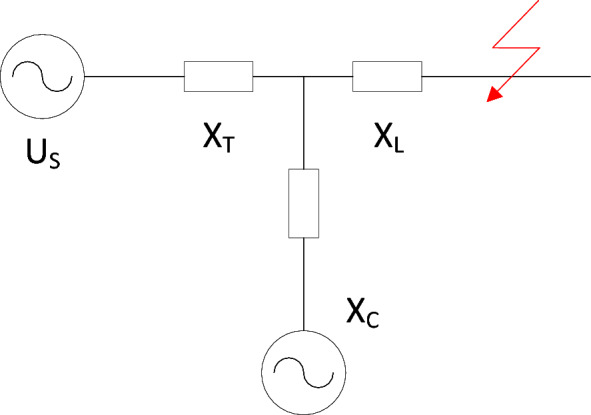
Equivalent diagram of a synchronous condenser connected to the power grid.

Figure 1 depicts the equivalent circuit when a synchronous condenser is connected to the power grid. The system’s equivalent power source is denoted as $U_s$, the network transfer impedance as $X_T$, the equivalent reactance of the synchronous condenser as $X_C$, and the equivalent reactance between the access point of the synchronous condenser and the short-circuit point as $X_L$.

When a short-circuit fault occurs before connecting the synchronous condenser, the per-unit value of the short-circuit current flowing through the fault point is expressed by Eq. (2):

2 $$\begin{aligned} I_d = \frac{1}{X_T + X_L} \end{aligned}$$

Conversely, when a short-circuit fault happens after the connection of the synchronous condenser, the per-unit value of the short-circuit current through the fault point is provided by E. (3):

3 $$\begin{aligned} I_d^{\prime } = \frac{1}{X_T + X_L // X_C} \end{aligned}$$

Since both the transmission line and stator windings of the synchronous condenser are inductive, $X_L > X_L / / X_C$ can be deduced. Consequently, $I_d^{\prime } > I_d$. According to the definition of short-circuit capacity, $S_d = \sqrt{3} \times U \times I_d$, the connection of the synchronous condenser results in increased short-circuit current and, accordingly, enhanced short-circuit capacity. Based on Eq. (1), elevating the short-circuit capacity leads to an improvement in MRSCR, thereby augmenting the renewable energy stations’ transmission limit.

## Time series production simulation optimization model

Time series production simulation is necessary to support system planning, medium and long-term power and electricity balance analysis, and quantitative analysis of renewable energy consumption. This paper takes the minimum system operation cost as the optimization goal. It considers the constraints such as power and electricity balance, unit output, reserve capacity, line capacity, Etc., to build a simulation optimization model of time series production.

### Objective function

The optimization objective is to minimize the total operational cost of the system, represented by Eq. (4):

4 $$\begin{aligned} \min C & =C_g\left( P_g^t\right) +C_{g s}+C_w\left( P_w^t\right) +C_{w d} P_{w d}^t+P_{b N} T_b C_{b 1}+\delta C_{b, \text{ in } }\left( P_{b, \text{ in } }^t\right) \\ & \quad + (1-\delta ) \cdot C_{b, \text{ out } }\left( P_{b, \text{ out } }^t\right) +C_{b 2}+C_{c 1}+2 \% \times S_c \times 8760 \times C_e+C_{c 2}+C_{l d} P_{l d}^t \end{aligned}$$

where $C_g\left( P_g^t\right)$ is the operating cost of the thermal power unit with an output power of $P_g^t$, $C_{g s}$ is the startup and shutdown cost of the thermal power unit, $C_w\left( P_w^t\right)$ is the operating cost for the renewable energy unit with an output power of $P_w^t$, and $C_{w d}$ is the renewable energy curtailment cost. Additional variables include $P_{w d}^t$ for curtailed renewable energy, $P_{b N}$ and $T_b$ for energy storage capacity and duration, $C_{b 1}$ for unit storage cost, $C_{b, \text{ in } }\left( P_{b, \text{ in } }^t\right)$ and $C_{b, \text{ out } }\left( P_{b, \text{ out } }^t\right)$ for the operating costs during charging and discharging. The calculation factor $\delta$ takes the value 1 during charging and 0 during discharging. Maintenance costs for energy storage and the synchronous condenser are represented by $C_{b 2}$ and $C_{c 2}$, respectively. Finally, $C_{l d}$ is the cost for load shedding and $P_{l d}^t$ is the amount of load shed at time $t$.

### Constraints

The model constraints can be divided into equality constraints and inequality constraints. Among them, equality constraints are system power and energy balance constraints and storage output constraints. Inequality constraints include conventional unit technical output constraints, renewable energy unit output constraints, system reserve capacity constraints, line transmission capacity constraints, energy storage charging and discharging power constraints, and energy storage state constraints.

1. Power balance constraint
  This ensures the power balance between input/output in the system at each time $t$.
  5 $$\begin{aligned} P_g^t+P_w^t-P_{w d}^t=P_l^t-P_{l d}^t \quad \forall t \in T \end{aligned}$$
  where $P_g^t$ and $P_w^t$ are the output of conventional thermal power and renewable energy units at time $t$, respectively. $P_{w d}^t$ is the curtailed renewable energy power, and $P_l^t$ and $P_{l d}^t$ are the load and the amount of load shedding at time $t$, respectively.
2. Energy storage output constraint
  This ensures the power balance between energy storage input/output at each time $t$.
  6 $$\begin{aligned} S_b^t=S_b^{t-1}+\left[ P_{b, \text{ in } }^t \cdot \eta _{b, \text{ in } }-\frac{P_{b, \text{ out } }^t}{\eta _{b, \text{ out } }}\right] \cdot \Delta t \end{aligned}$$
  where $S_b^t$ and $S_b^{t-1}$ are the remaining capacities at times $t$ and $t-1$, respectively. $P_{b, \text{ in } }^t$ and $P_{b, \text{ out } }^t$ are the charging and discharging powers, respectively. $\eta _{b, \text{ in } }$ and $\eta _{b, \text{ out } }$ are the efficiencies during charging and discharging, respectively. $\Delta t$ is the time interval.
3. Conventional thermal power output constraints
  Due to technological and economic limitations, the thermal power units have specific minimum and maximum output levels.
  7 $$\begin{aligned} & 0 \le P_{g \text{ min } } \le P_g^t \le P_{g \max } \quad \forall t \in T \\ & \quad -\Delta P_g^{\text{ down } } \le P_g^t-P_g^{t-1} \le \Delta P_g^{u p} \quad \forall t \in T \end{aligned}$$
  where $P_{g \min }$ and $P_{g \max }$ are the minimum and maximum technical outputs of the thermal power units. $\Delta P_g^{\text{ down } }$ and $\Delta P_g^{\text{ up } }$ are the rates of decrease and increase in power output, respectively.
4. Renewable energy units output constraints
  The predicted output value of renewable energy in any time $t$ is the sum of the actual output and the cut-off power.
  8 $$\begin{aligned} \left\{ \begin{array}{cc} P_w^t+P_{w d}^t=P_{w \Sigma }^t &{} \forall t \in T \\ P_w^t \ge 0, P_{w d}^t \ge 0 &{} \forall t \in T \end{array}\right. \end{aligned}$$
  where $P_{w \Sigma }^t$ is the predicted output value of renewable energy at time $t$.
5. System standby constraints
  9 $$\begin{aligned} {} & (1+\alpha ) P_l^t-P_{l d}^t \le P_{g \max }^t+P_{w \Sigma }^t \quad \forall t \in T \\ & P_{g \text{ min } }^t \le (1-\beta ) P_l^t-P_{l d}^t \quad \forall t \in T \end{aligned}$$
  where $\alpha$ and $\beta$ are the positive and negative reserve rates of the system, respectively.
6. Line capacity constraint
  10 $$\begin{aligned} 0 \le S_r^t \le S_{r \max } \quad \forall t \in T \end{aligned}$$
  where $S_r^t$ is the line transmission capacity at time $t$ and $S_{r \max }$ is the maximum line capacity.
7. Charge discharge power constraints
  11 $$\begin{aligned} \begin{aligned}{}&0 \le P_{b, \text{ in } }^t \quad \forall t \in T \\&P_{b, \text{ out } }^t \le P_{b N} \quad \forall t \in T \end{aligned} \end{aligned}$$
  where $P_{b N}$ is the installed capacity of the energy storage system.
8. Energy storage level constraints
  12 $$\begin{aligned} \begin{aligned}{}&0 \le S_b^t \le P_{b N} \cdot T_b \quad \forall t \in T \\&S_b^{t=0}=S_b^{t=T_b}=\mu _b \cdot P_{b N} \cdot T_b \quad \forall t \in T \end{aligned} \end{aligned}$$
  where $T_b$ is the energy storage duration and $\mu _b$ is the percentage of initial energy storage level.

## Optimization methods

**Figure 2:**
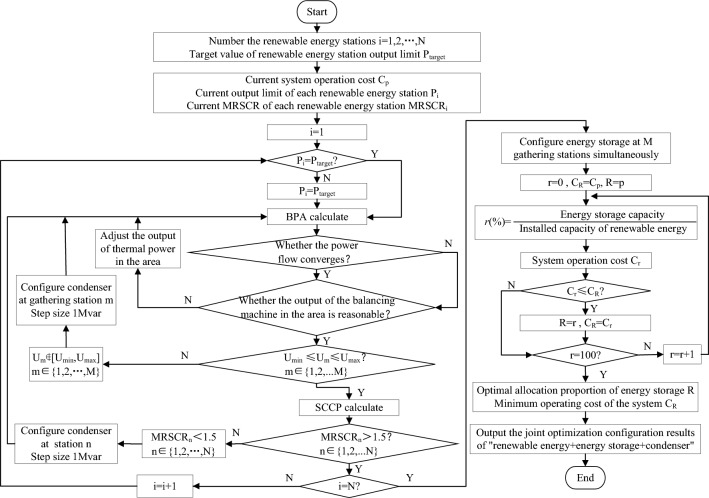
Methodology flow chart.

As mature software for power system analysis, BPA and SCCP are widely used in power system planning and design, dispatching operations, teaching, and scientific research departments. Combining the software advantages of BPA and SCCP, this research proposes a time series production simulation optimization method considering the joint optimization configuration of “renewable energy + energy storage + synchronous condenser.” The method flow chart is shown in Fig. 2.

The basic steps are as follows.

1. Number the renewable energy station. Specify the requirements of renewable energy stations for sending out and consuming, and determine the target value of renewable energy station output limit.
2. Judge whether the current output value of each station meets the delivery and consumption requirements one by one. If not, the output value of the station shall be increased to the target output value, and then the BPA program shall be run.
3. After the BPA calculation is completed, check whether the power flow converges and whether the output of the balancing machine in the area is reasonable. If the power flow does not converge and the output of the balancing machine exceeds the reasonable range, the output of the thermal generator set in the regional power grid shall be adjusted to adapt to the change in the renewable energy output.
4. Check whether the bus voltage of the renewable energy gathering station is within a reasonable range. Select the gathering station m that does not meet the voltage requirements, and configure synchronous condensers in this station with a step of 1Mvar. Return to run the BPA program.
5. When the power flow converges, and the output of the balancing machine and the bus voltage of the converter station are reasonable, run the SCCP program. After the SCCP calculation, check whether the MRSCR of all renewable energy stations meets the requirement of more than 1.5. Select station j with a short circuit ratio less than 1.5, and configure synchronous condensers in this station with a step of 1Mvar. Return to the BPA program until the MRSCR of all renewable energy stations is above 1.5.
6. All renewable energy gathering stations are equipped with energy storage at the same time. The configured energy storage capacity is proportional to the installed capacity of renewable energy under the gathering station, with a step of 1%. Through cycle optimization, the optimal allocation ratio R of energy storage and the lowest operating cost of the system is obtained.
7. Output the optimal joint configuration scheme of “renewable energy + energy storage + synchronous condenser.” The program ends.

## Case study

### Case introduction

**Figure 3:**
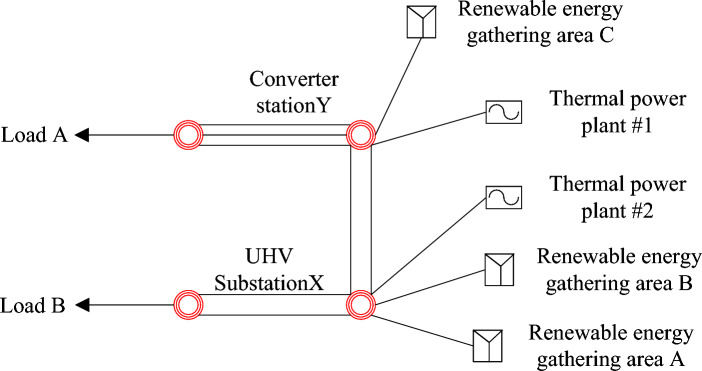
Schematic diagram of test system structure.

This study investigates the same case scenarios modeled in^29–31^. It focuses on a distantly located energy base that utilizes a wind + thermal power combination. Electricity from this combined source is transmitted through a single channel that incorporates AC and DC technologies, interconnected via a converter station. The system structure and its details are illustrated in Fig. 3. The source side of this model includes two thermal power plants (#1, #2) and three renewable energy gathering areas (A, B, C). Each gathering area contains a renewable energy gathering station and multiple wind farms. Gathering stations A and B and thermal power plant #2 are connected to UHV substation X while gathering station C and thermal power plant #1 are connected to converter station Y. These substations and converter stations are interconnected through double circuit lines. The converter station connects to load A via an ultra-high voltage DC channel with a transmission limit of 6000MW, and the UHV AC channel is connected to load B with a transmission limit of 2600MW. The research explores the impact of an optimized configuration involving “renewable energy + energy storage + synchronous condenser” on the consumption and dispatch of renewable energy.

The average renewable energy output in the original state of the three renewable energy gathering areas is 0.40, 0.56, and 0.47, respectively. The optimization goal is to improve the stable transmission limit of renewable energy stations to 70% on the premise of meeting the MRSCR higher than 1.5 and the safe and stable operation of the system. The time series production simulation optimization method proposed in Chapter 3 is used to jointly optimize the configuration of the test system with “renewable energy + energy storage + synchronous condenser.”

### Renewable energy consumption and economic analysis

**Figure 4:**
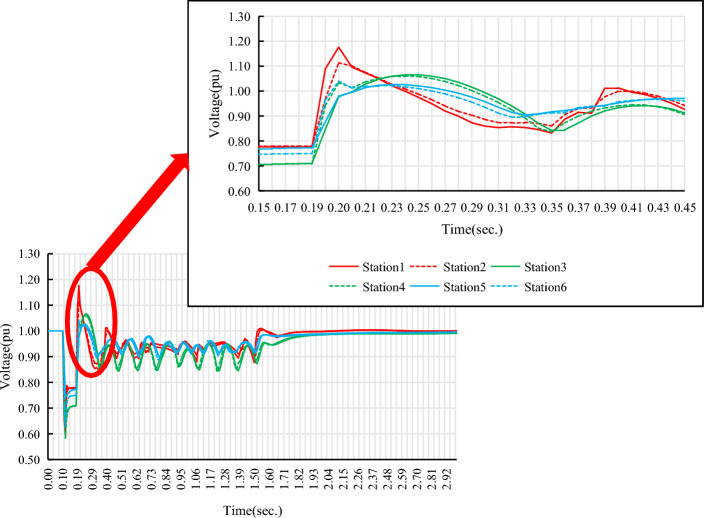
Overvoltage at the renewable energy generator terminal during AC dault disturbance.

**Figure 5:**
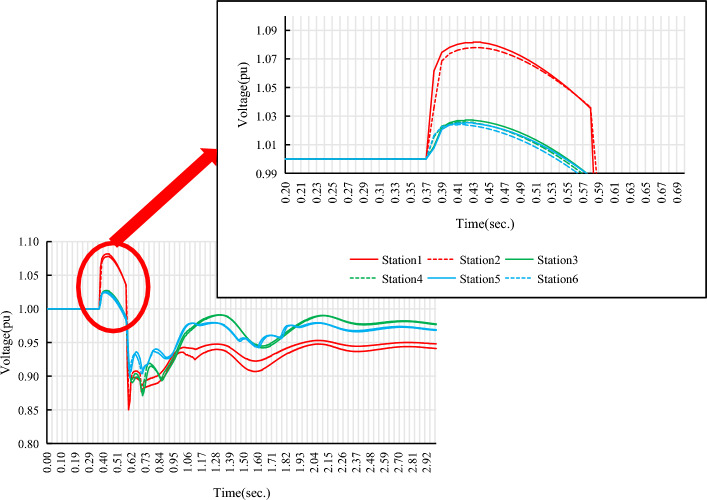
Overvoltage at the renewable energy generator terminal during DC fault disturbance.

Select the renewable energy stations with the highest and lowest MRSCR in each renewable energy gathering area as the research objects, and use BPA simulation software to analyze the relationship between the MRSCR of the renewable energy station connection point and the overvoltage of the renewable energy machine terminal during the AC fault (single-phase ground transient fault) and DC fault (DC bipolar lock fault) disturbance of the testing system. The simulation results are shown in Table 2, Figs. 4, and 5.

**Table 2:** Transient stability simulation results.

Renewable energy gathering area	Renewable energy station	MRCSR	Renewable energy terminal overvoltage (p.u.)	
		1.735	AC fault	DC fault
A	1	1.988	0.178	0.082
	2	1.988	0.117	0.080
B	3	1.588	0.065	0.027
	4	1.757	0.062	0.026
C	5	1.724	0.040	0.026
	6	1.906	0.026	0.024

From Figs. 4 and 5, it can be seen that during the AC/DC fault disturbance period in the power system, the overvoltage at the renewable energy generator terminal is related to MRSCR. In the same renewable energy gathering area, the larger the MRSCR of the renewable energy station connection point, the smaller the overvoltage at the renewable energy terminal. On the contrary, the smaller the MRSCR of the renewable energy station connection point, the greater the overvoltage at the renewable energy machine terminal.

### Renewable energy consumption and economic analysis

The optimized configuration results of “renewable energy + energy storage + synchronous condenser” of the test system are shown in Table 3 below.

**Table 3:** Optimized configuration results.

Renewable energy gathering area	Configured capacity of synchronous condenser	Proportion of energy storage configuration (%)
A	Renewable energy station 1740 Mvar	20
	Renewable energy gathering Station: 2160 Mvar	
B	Renewable energy station 240 Mvar	20
	Renewable energy gathering Station: 720 Mvar	
C	Renewable energy station 120 Mvar	20
	Renewable energy gathering Station: 720 Mvar	
	5700 Mvar	20

**Figure 6:**
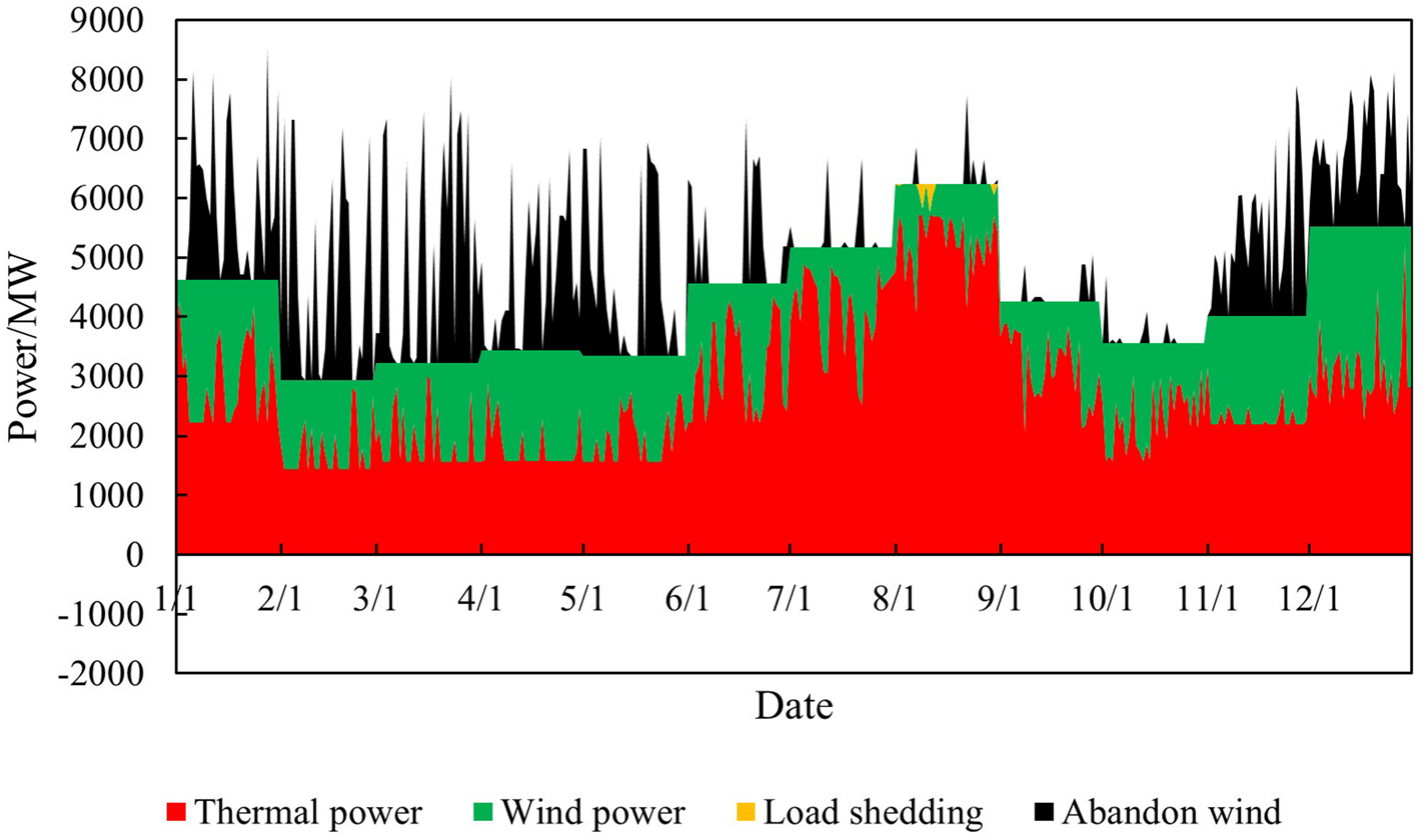
Time series production simulation results before renewable energy output increase.

**Figure 7:**
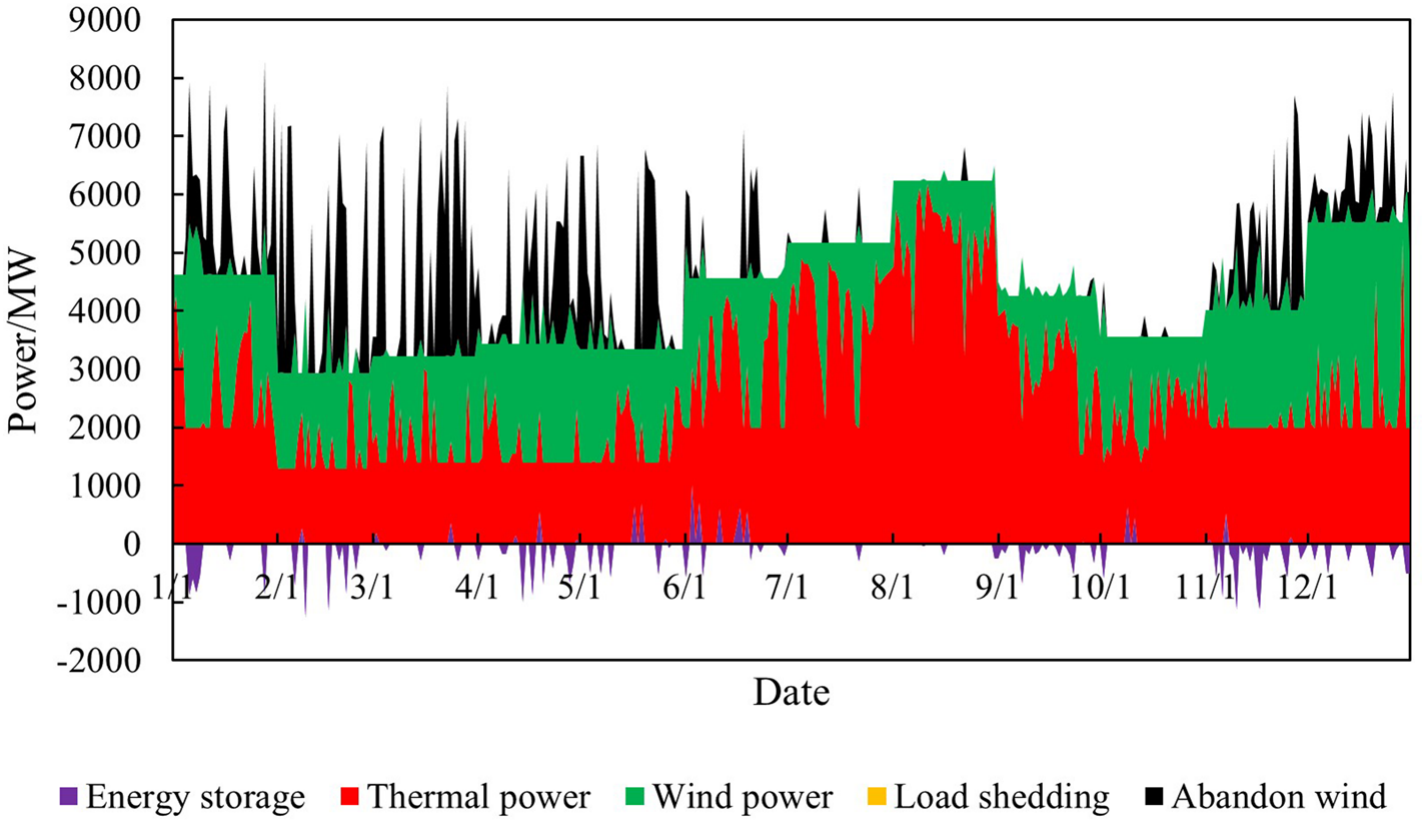
Time series production simulation results after renewable energy output increase.

Input the renewable energy output curve parameters (8760 points) into the model established in Chapter 2 to obtain the simulation diagram of the overall system operation before and after the renewable energy output is increased, as shown in Figs. 6 and 7.

1. Renewable energy consumption analysis
  The situation of wind power generation after the increase in renewable energy output is shown in Fig. 8. Before the increase in renewable energy output, the wind power generation capacity was $1.31 \times 10^7$  MWh, and the utilization hours were 1873h. Abandon wind was $6.27 \times 10^6$ MWh, and the abandonment rate was 32.37%. After the increase in renewable energy output, the wind power generation capacity is $1.53 \times 10^7$ MWh, and the additional power generation capacity is $2.18 \times 10^6$ MWh. Among them, $5.30 \times 10^5$ MWh are added due to the allocation of energy storage, accounting for 24.34% of the total added electricity. Additional $1.65 \times 10^6$ MWh are generated due to the configuration of the synchronous condenser, accounting for 75.66% of the total additional electricity generated. The utilization hours of wind power are 2184h, an increase of 311h. Abandon wind is $4.01 \times 10^6$ MWh, and the abandonment rate is 21.13%. According to the results, the “renewable energy+ energy storage+ synchronous condenser” mode can effectively improve the utilization rate of renewable energy, reduce wind curtailment, and promote the consumption of renewable energy.

**Figure 8:**
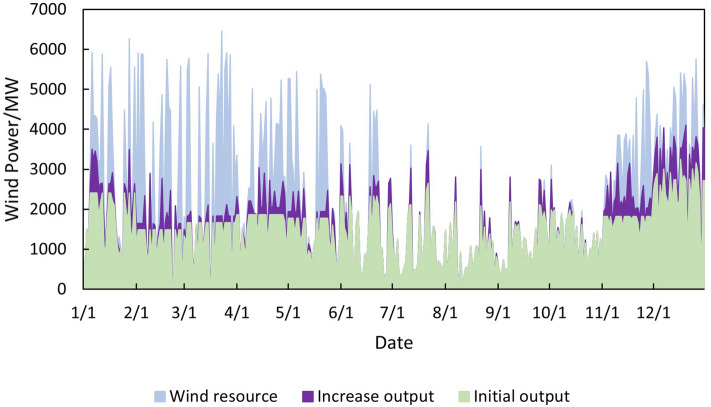
Wind power generation after the increase of renewable energy output.
2. Thermal power output analysis
  Before and after the increase in renewable energy output, the output of thermal power units was $2.85 \times 10^7$ MWh and $2.70 \times 10^7$ MWh, respectively, reducing $1.50 \times 10^6$ MWh. The utilization hours were reduced from 3907h to 3699h. When the system is not restricted by the channel and the load has sufficient absorptive capacity, the system will select renewable energy with lower operating costs and cleaner energy to meet the load demand, thereby reducing the utilization hours of conventional units.
3. Load shedding analysis
  Before the renewable energy output was increased, the system load shedding was $6.50 \times 10^5$ MWh, accounting for 1.54% of the total load. After the renewable energy output is increased, the system load shedding is $2.12 \times 10^4$MWh, accounting for 0.05% of the total load. Load shedding mainly occurs in August because August is the peak load period, and the wind power output decreases due to the climate. At the same time, the cost of thermal power reserve for system startup is higher than the cost of load shedding, so partial load shedding is used to meet the power and electricity balance of the system.
4. Typical daily analysis
  Take March 15th as an example, and the daily load is $9.21 \times 10^4$ MWh. Before the renewable energy output was increased, thermal power output was $5.05 \times 10^5$MWh, wind power output was $4.16 \times 10^4$MWh, the abandoned wind was $1.10 \times 10^4$ MWh, the abandonment rate is 20.85%, and the simulation results of time series production are shown in Fig. 9. After the optimized configuration of “renewable energy + energy storage + synchronous condenser,” the renewable energy output limit is increased, and the simulation results of time series production are shown in Fig. 10. Total wind power output is $4.89 \times 10^4$ MWh. Among them, $4.35 \times 10^3$ MWh are added due to the allocation of energy storage. Additional $2.91 \times 10^3$ MWh are generated due to the configuration of the synchronous condenser-no abandoned wind. Thermal power output is $4.34 \times 10^4$ MWh, decreased by 0.71 MWh. The change in wind power output is shown in Fig. 11.
5. Economic analysis
  The total system operation cost includes thermal power, wind power, energy storage, synchronous condensers, and load shedding costs. After the increase in renewable energy output, the total system operation cost changed from $2.44 \times 10^{10}$ yuan to $1.60 \times 10^{10}$ yuan, a decrease of $8.40 \times 10^9$ yuan, as shown in Fig. 12. The thermal power cost has changed from $9.54 \times 10^9$ yuan to $9.11 \times 10^9$ yuan, a decrease of $4.30 \times 10^8$ yuan. The load shedding cost has changed from $1.30 \times 10^{10}$ yuan to $4.24 \times 10^8$ yuan, a decrease of $1.26 \times 10^{10}$ yuan. The additional electricity generated by wind power is $2.18 \times 10^6$ MWh. If the electricity price is calculated at 0.47 yuan/kWh, the economic income will be increased by $1.02 \times 10^9$ yuan in advance. The energy storage cost is increased by $7.40 \times 10^8$ yuan. The synchronous condenser cost is increased by $3.94 \times 10^9$ yuan.

**Figure 9:**
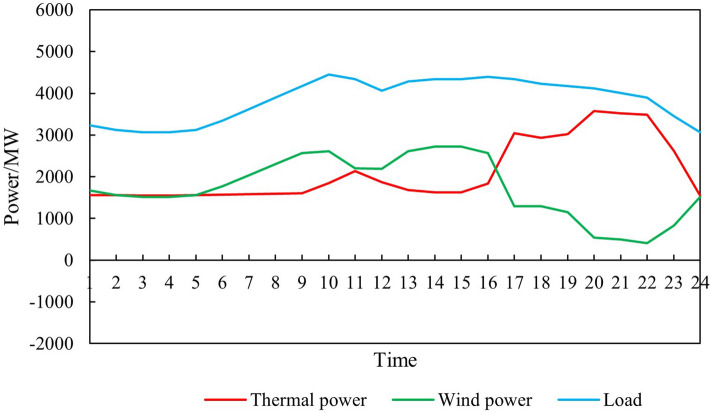
Time series production simulation results before renewable energy output increase (March 15th).

**Figure 10:**
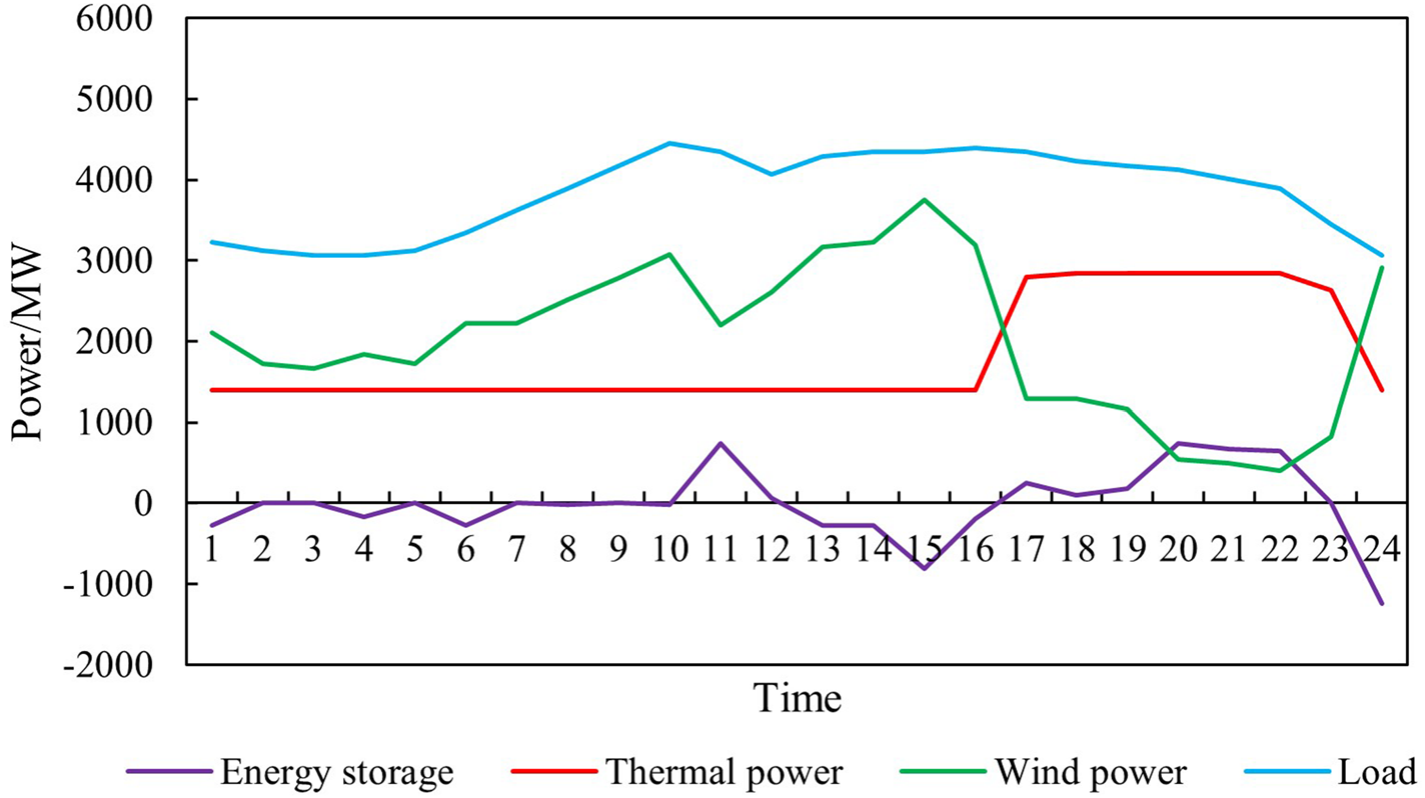
Time series production simulation results after renewable energy output increase (March 15th).

**Figure 11:**
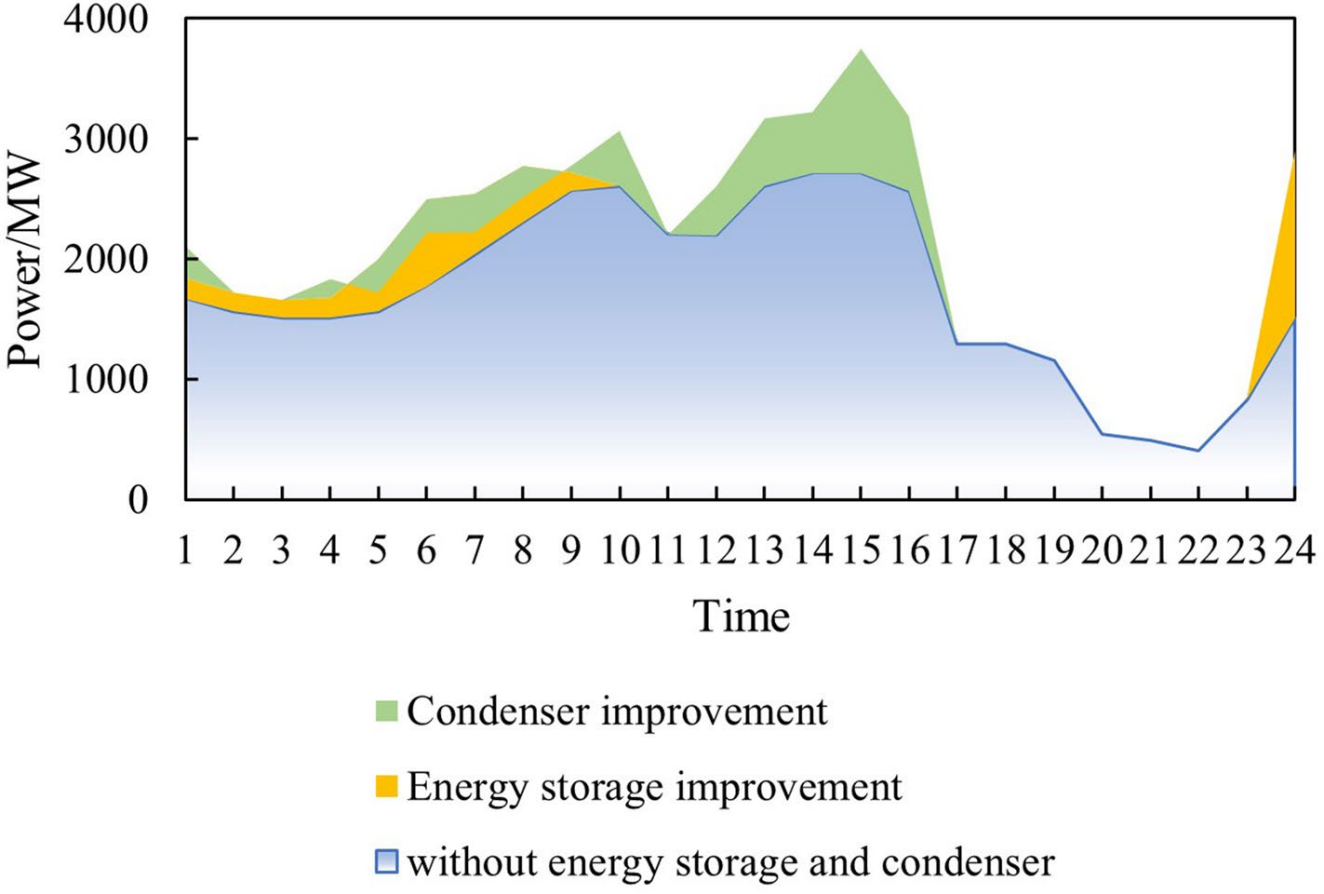
Change of wind power output.

**Figure 12:**
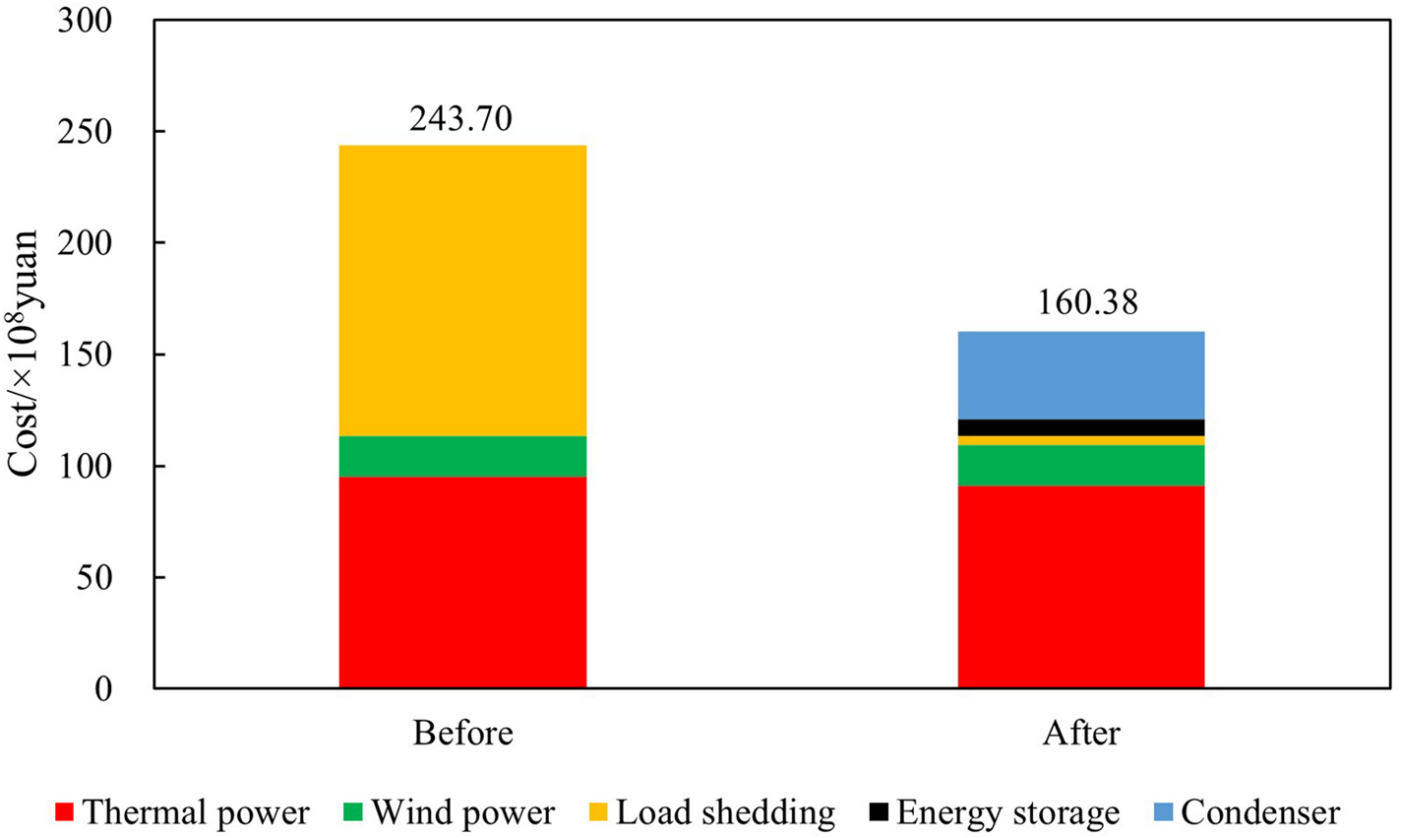
Change of total system operation cost.

## Conclusions

Two pivotal conclusions are drawn in this paper. (1) Introducing synchronous condensers in renewable energy stations effectively enhances the MRSCR and bolsters the system’s voltage support capacity. This not only elevates the delivery limit of renewable energy stations but also fosters renewable energy consumption. A reasonable allocation of energy storage ensures the safety support of thermal power for system operation and reduces the operational hours of thermal power units. This mechanism contributes to solving the issue of large-scale renewable energy curtailment. The “Renewable Energy + Energy Storage + Synchronous Condenser” joint intelligent control and optimization technology effectively increases the renewable energy transmission capacity limit, enhances the utilization rate of renewable energy, and meets the renewable energy transmission and consumption requirements. (2) “Renewable energy + energy storage + synchronous condenser” joint intelligent control and optimization technology can effectively reduce the system operation cost and improve the system operation economy. The time series production simulation optimization method proposed in this paper considering the joint optimization configuration of “renewable energy + energy storage + synchronous condenser” can provide scientific decision support for investors.

## Data Availability

The data that support the findings of this study are available from [China Electric Power Research Institute] but restrictions apply to the availability of these data, which were used under license for the current study, and so are not publicly available. Data are however available from the authors upon reasonable request and with permission of [China Electric Power Research Institute].
